# Supplementary material for: Resveratrol Improves the Digestive Ability and the Intestinal Health of Siberian Sturgeon
Source: Int J Mol Sci. 2022 Oct 9;23(19):11977. doi: 10.3390/ijms231911977 (PMC9569792; doi:10.3390/ijms231911977)
Supplement: Supplementary file 1 [file ijms-23-11977-s001.zip › ijms-1957950-supplementary.pdf]

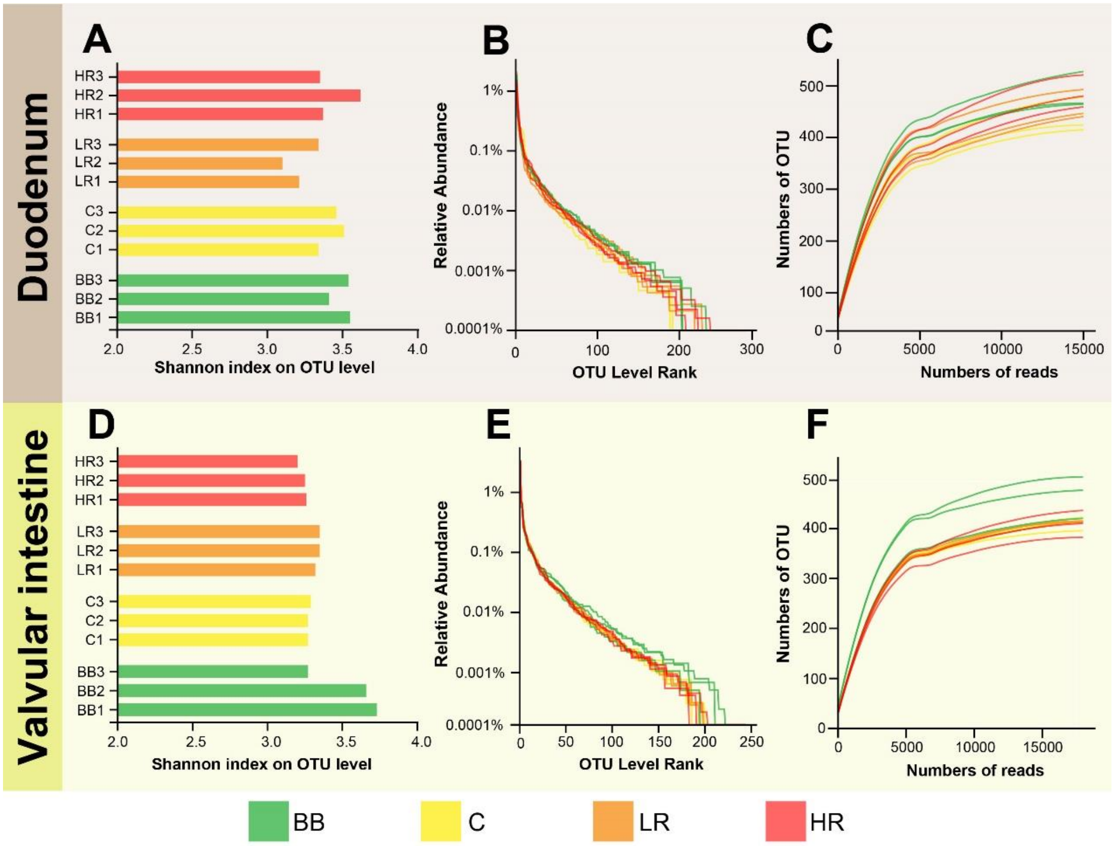

Figure S1 Species annotation and assessment in the duodenum and the valvular intestine. A and D: Shannon index of each replicate at OTU level. B and E: Rank-Abundance curve at OTU level. C and F: Dilution curve at OTU level.

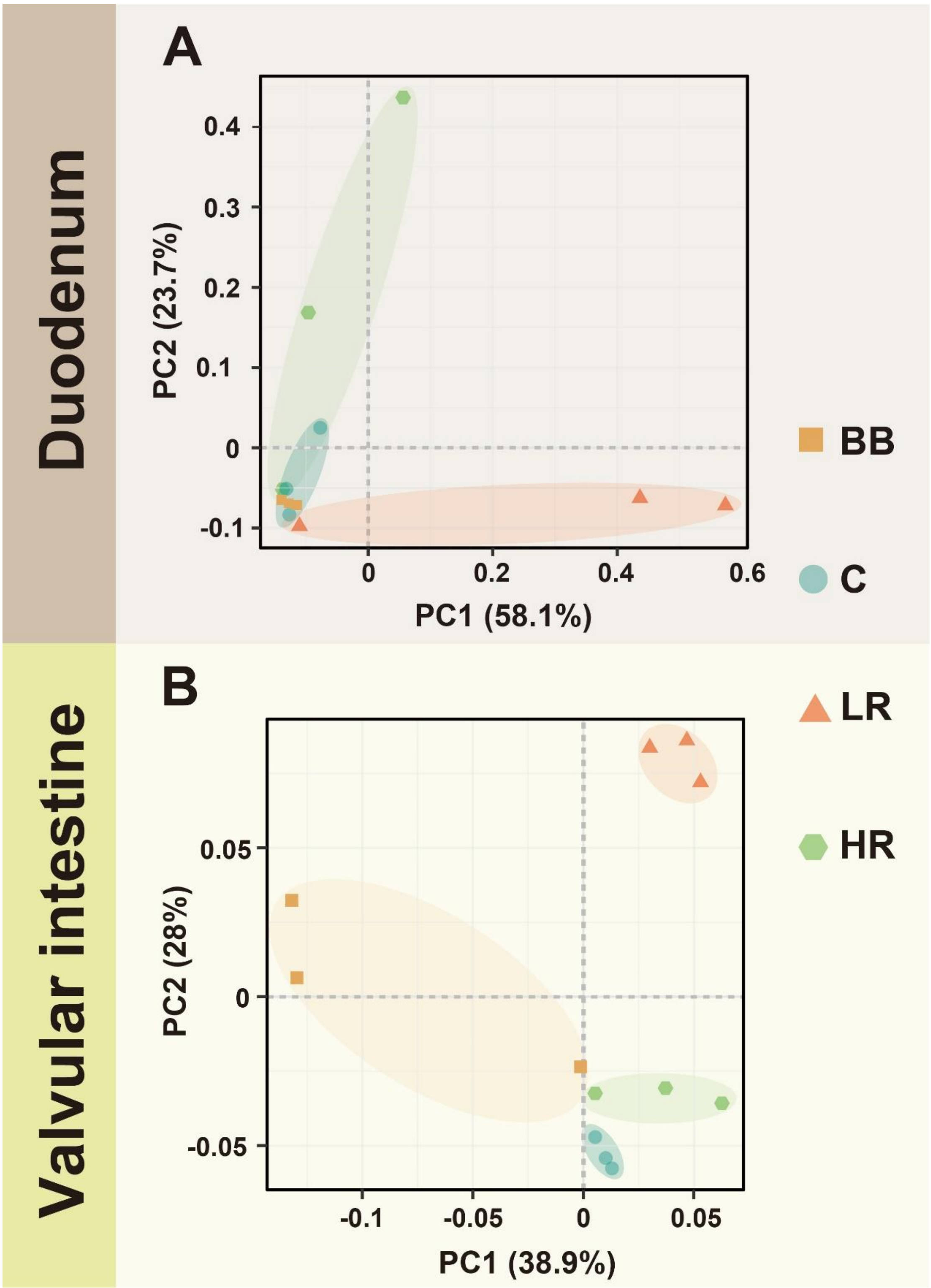

Figure S2 Principal coordinates analysis (PCoA) plots based on Bray-Curtis dissimilarity of 16S rRNA amplicon sequencing of different groups in the duodenum (A) and valvular intestine (B), each color corresponds to a different group.

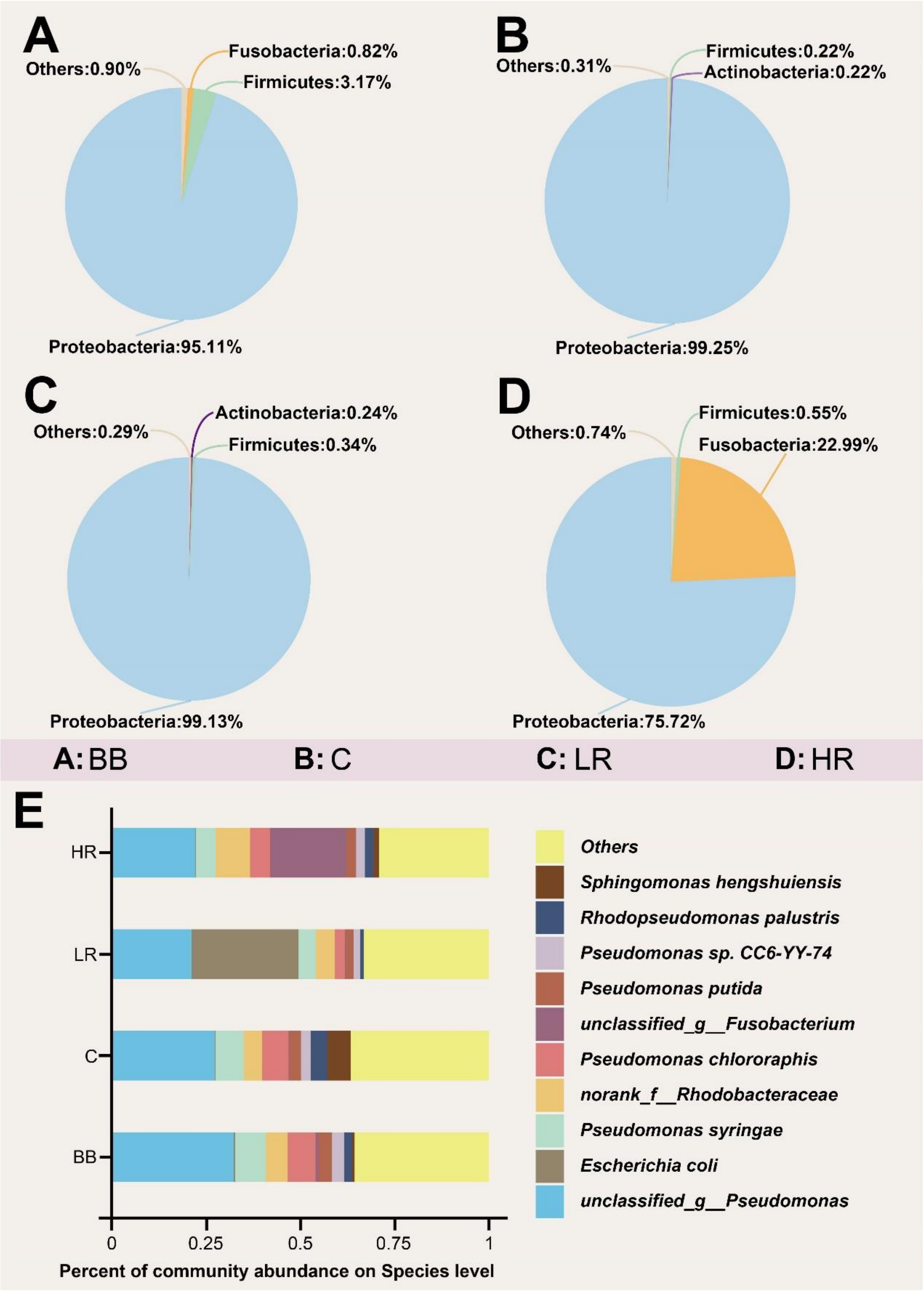

Figure S3 Comparison of microbial community structures among different groups in the duodenum. A-D: Pie charts of the most abundant phyla. E: Top ten microorganisms at species level.

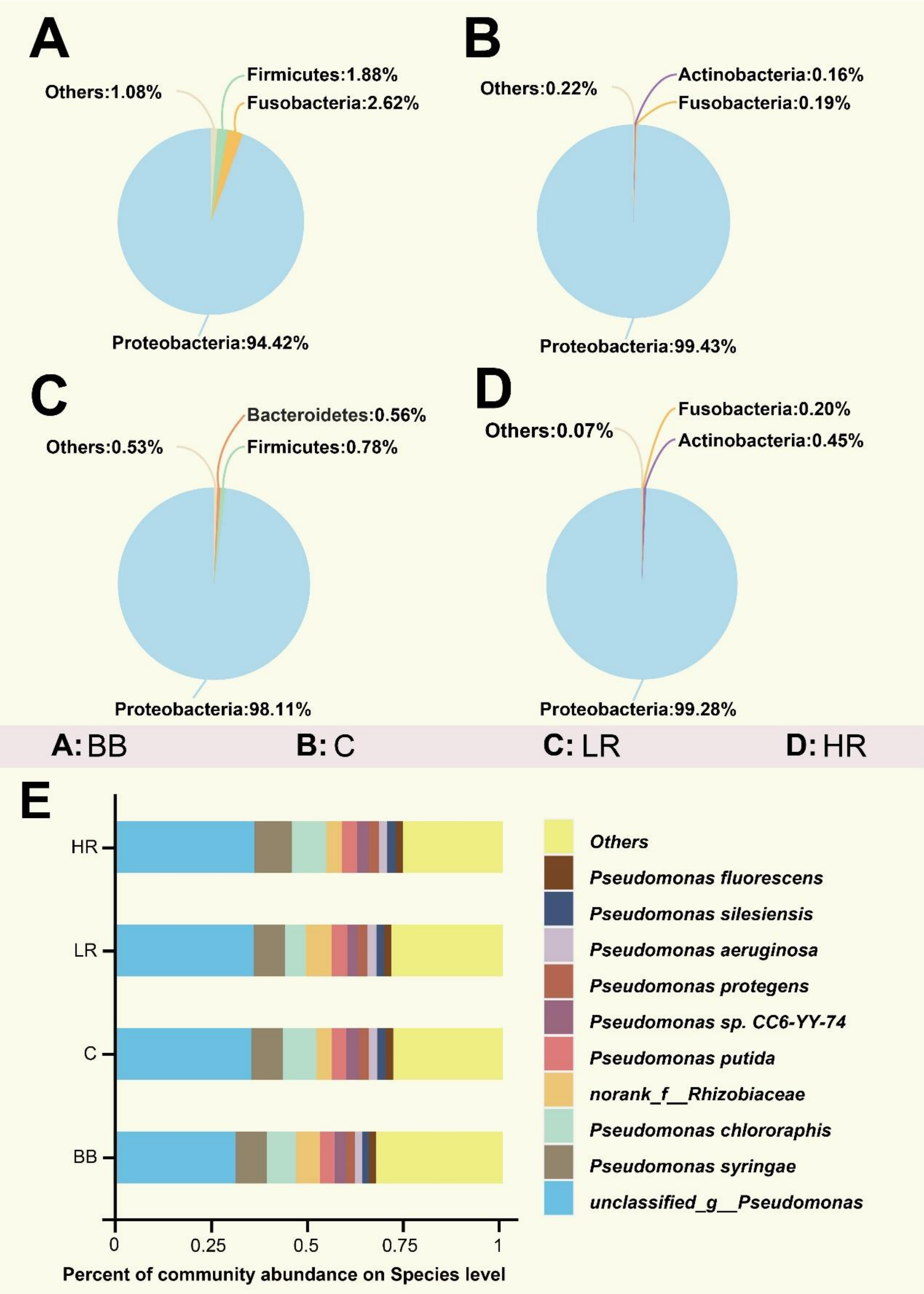

Figure S4 Comparison of microbial community structures among different groups in the valvular intestine. A-D: Pie charts of the most abundant phyla. E: Top ten microorganisms at species level.

Table S1. Alpha diversity of intestine communities.

Organ	Sample category	Replicate	Raw _Num	Clean _Num	Ave_length	Chao	Shannon	Simpson	Coverage
Duodenum	Before breeding	Du_BB1	31,556	16,114	1,432	326.88	3.56	0.90	0.9977013
		Du_BB2	39,258	22,722	1,417	317.12	3.41	0.87	0.9977634
		Du_BB3	38,394	20,087	1,420	474.02	3.51	0.88	0.9939115
	Control group	Du _C1	30,906	24,362	1,430	304.23	3.34	0.88	0.9970179
		Du _C2	32,880	24,651	1,421	432.61	3.53	0.91	0.9941600
		Du _C3	42,870	33,114	1,408	294.97	3.47	0.93	0.9971421
	Low resveratrol	Du _LR1	32,647	19,201	1,417	335.90	3.25	0.87	0.9958996
		Du _LR2	36,718	23,784	1,402	358.33	3.11	0.84	0.9960239
		Du _LR3	32,532	18,642	1,399	431.12	3.38	0.88	0.9949677
	High resveratrol	Du _HR1	35,173	28,170	1,401	333.69	3.35	0.90	0.9958996
		Du _HR2	40,651	31,442	1,404	422.00	3.65	0.92	0.9943464
		Du _HR3	56,878	45,070	1,403	431.00	3.36	0.91	0.9939737
Valvular intestine	Before feeding	Vi_BB1	39,720	27,411	1,406	438.16	3.73	0.90	0.9970080
		Vi_BB2	35,440	23,985	1,417	527.84	3.66	0.90	0.9941210
		Vi_BB3	30,752	24,264	1,422	306.57	3.27	0.87	0.9976904
	Control group	Vi_C1	37,258	24,258	1,416	315.92	3.27	0.87	0.9974804
		Vi_C2	33,813	24,597	1,409	257.89	3.27	0.87	0.9987402
		Vi_C3	33,845	19,345	1,418	331.50	3.29	0.87	0.9969555
	Low resveratrol	Vi_LR1	34,031	19,601	1,409	383.91	3.32	0.87	0.9963781
		Vi_LR2	34,530	20,797	1,402	374.17	3.35	0.86	0.9969555
		Vi_LR3	33,071	19,101	1,418	353.64	3.35	0.87	0.9974804
	High resveratrol	Vi_HR1	43,620	33,846	1,414	271.50	3.26	0.87	0.9983203
		Vi_HR2	35,172	26,858	1,416	379.30	3.25	0.86	0.9962732
		Vi_HR3	36,084	28,996	1,416	316.84	3.20	0.86	0.9972705
